# Challenges to the provision of home care and support for people with severe mental illness: Experiences and perspectives of patients, caregivers, and healthcare providers in Dar es Salaam, Tanzania

**DOI:** 10.1371/journal.pgph.0001518

**Published:** 2023-01-24

**Authors:** Joel Seme Ambikile, Masunga K. Iseselo

**Affiliations:** Department of Clinical Nursing, Muhimbili University of Health & Allied Sciences (MUHAS), Dar es Salaam, Tanzania; The University of Texas Health Science Center at Houston School of Public Health - San Antonio Campus, UNITED STATES

## Abstract

A balance between hospital-based and community-based services is needed to effectively provide mental health services for people with mental illness. As an essential part of community mental health services, home-based care plays an important role in meeting patients’ needs, and should, therefore, be appropriately provided. To achieve this, there is a need to understand the challenges faced and take relevant actions to address them. This study aimed to explore challenges to home care and support for people with mental illness in Temeke district, Dar es Salaam. We used a descriptive qualitative study approach to explore challenges to home care and support for people with mental illness among patients, their caregivers, and healthcare providers. The purposeful sampling method was used to recruit participants at Temeke hospital, data was collected using in-depth interviews and focus group discussions, and analysis was performed using a content analysis framework. Four main themes highlighting challenges encountered in the provision of home care and support for individuals with mental illness were revealed. They include poor understanding of mental illness, abandonment of patients’ care responsibilities, disputes over preferred treatment, and lack of outreach services for mental health. Participants also provided suggestions to improve home care and support for people with mental illness. Home care for people with mental illness is affected by poor knowledge of the mental illness, social stigma, and lack of outreach visits. There is a need for the provision of health education regarding mental illness, stigma reduction programs, and funding and prioritization for outreach home visits to improve home care and support for people with mental illness. Further research is needed to determine the magnitude of these challenges and factors that can facilitate the provision of support in similar settings.

## Introduction

Mental health care services may be provided in two main settings namely institutional-based and community-based [1]. In the 19^th^ and early 20th centuries, psychiatric hospitals served as the main institution of mental healthcare for individuals with severe mental illness unlike nowadays where the emphasis is placed on community-based services as a result of mental health policy reforms that have occurred [2]. It has been noted that centralized and institutionalized mental health care relies mainly on scarce specialist manpower and creates a major treatment gap for patients and places an unnecessary burden on the individual, their family, and society [3]. De-institutionalization of mental health services was meant to provide treatment and rehabilitation of the severely mentally ill within the community and the promotion of mental health generally [4]. However, for various reasons, many mental health services remain too hospital-centric, often without adequate outreach services [5]. Moreover, there is no strong evidence that a comprehensive system of mental healthcare can be provided by either hospital-based care or community-based services, and the tendency is rather to have a balance that includes both components [6, 7]. Home-based care is an important part of community mental health services.

It has been long known that home-based care for people with serious mental illness has superior outcomes compared to hospital-based care as it improves symptoms management and social adjustment and leads to patients’ and relatives’ satisfaction [8]. It enables both the patient and his or her family to stay at their houses and not be bothered with a long-term stay in institutional-based care and minimizes the burden on outpatient services [9]. When home care is supported by home visits and support by healthcare workers and taking responsibility for both health and social care needs, it reduces admission days in the hospital and costs associated with care [10]. This is because patients and caregivers are given information regarding the identification of psychiatric symptoms, medication administration and management of side effects, disease process, and basic skills for managing mental health symptoms. This practice helps the patients to comply with the prescribed treatment. Therefore, supported home care is associated with stabilization of disease conditions, improved daily living abilities, enhancement of communication ability, and generally improved quality of life [11].

Like other Low- and Middle-Income Countries (LMICs), the burden of mental disorders in Tanzania is high. For instance, disability-adjusted life years (per 100,000 population) is 2,728 and suicide mortality rate (per 100,000 population) is 5.4. Total mental health expenditure per person in the country is estimated to be Tanzanian Shillings 43.1. The government spends only 4% of the total government health expenditure on mental health. The country has 2 mental hospitals, 5 psychiatric units in general hospitals, one forensic inpatient unit, and 4 residential care facilities. The total number of the mental health workforce is 278 with rate per 100,000 populations being 0.06 for psychiatrists, 0.36 for mental health nurses, 0.01, for psychologists, 0.06 for social workers, and 0.02 for occupational therapists [12]. Various barriers to effective provision of mental health services that affect LMICs also occur in Tanzania. They are mainly attributed to poor governance [13, 14] which lead to human and nonhuman resource for mental health challenges such as poor infrastructure and inadequate medications [13, 15, 16]. These make accessibility of mental health services difficult coupled with presence of very few psychiatrists who are mostly based in big cities and are often not accessible, especially by the rural populations [17, 18]. Therefore the magnitude of mental health problems and treatment gap in the country is high and due to limited resources, the government is unable to meet the vast mental health needs of its population [19, 20].

Home care for people with mental illness may be associated with certain challenges. These include high burden of care, social exclusion, health impact, not meeting the needs of caregivers, burnout (emotional distress), high social stigma, lack of support networks (low social support for caregivers), and low quality of life of caregivers [21, 22]. Sometimes caring for patients with serious mental illness at home may be difficult and unbearable due to violence perpetrated by the patients and safety concerns and the lack of skills required to manage patients that would make caregivers wish patients were kept in institutions [23]. Such challenges are influenced by the dynamics of family relationships, cultural and spiritual context of the understanding of mental illness, caregiving practices, and community attitudes [24]. To successfully meet the needs of people with mental illness, it is imperative to understand the challenges faced in the provision of home care. Therefore, this study aimed to explore challenges to home care and support for people with mental illness in Temeke district, Dar es Salaam, which were achieved through participants’ interviews.

## Materials and methods

### Study setting

This study was conducted at Temeke regional referral hospital, in Dar es Salaam, Tanzania. Temeke is one of the five districts constituting Dar es Salaam city which was selected for the study. Dar es Salaam is a very fast-growing and largest commercial city in Tanzania with a population of around seven million [25]. Like other districts in the city, Temeke has socio-economic characteristics likely to influence the occurrence of mental health problems/disorders. Both inpatient and outpatient services are provided at Temeke regional referral hospital including mental health services which is part of the outpatient department. During the period of data collection, the outpatient department received between 1,000 and 1,500 patients a day attending various service delivery points, and more than 400 patients with mental health problems were seen every month.

### Study design

The design of the current study was qualitative descriptive. A qualitative descriptive design emphasizes the study of ascribed meaning from participants and adopts a naturalistic approach to comprehend the event in a natural setting [26]. Such an approach can provide a rich account of a phenomenon that is poorly understood [27]. Qualitative descriptive research seeks to understand the experience, occurrences, and interactions of a phenomenon from the perspective of those who are closest to it [26]. Because this study aimed to identify the challenges faced in the provision of home care and support for people with mental illness, a qualitative descriptive design was particularly relevant.

### Study participants

Participants involved in this study were patients with mental illness, caregivers of mentally ill patients, and healthcare providers. Patients with mental illness were those who were attending a clinic at the Mental Health Unit, caregivers were those who escorted patients with mental illness to the clinic, and healthcare providers were those attending patients with mental illness at the clinic including the district mental health coordinator.

### Inclusion and exclusion criteria

The inclusion criteria were; patients who had a chronic mental illness; caregivers who had stayed with a mentally ill patient for at least six months; and healthcare providers who had worked in the mental health section for at least six months. The exclusion criteria were; mentally unstable patients (those who had psychotic symptoms and disorganized thinking) since they could not provide useful information during the interview; caregivers who had very serious patients who needed constant attention and could thus not leave them; and healthcare providers who were not available due to other important responsibilities.

### Sampling methods and procedure

A purposeful sampling procedure was applied to get all participants, i.e., patients, healthcare providers, and caregivers. The decision to use this sampling method was based on information-rich participants who could be willing to share their concepts and views about challenges in caring for patients with mental illness. Research assistants who were also nurses working at mental health clinics recruited the participants on clinic days for mental health services. For patients, the research assistant provided a list of people with mental illness who attended the clinic on that particular day for follow-up. The list was reviewed to identify those who met the inclusion criteria and asked for an interview. Potential participants were selected depending on their mental health status and their ability to provide information. On the other hand, all healthcare providers who met the inclusion criteria were invited to participate in the study. Caregivers who came with the patient to the follow-up clinic and met the inclusion criteria were involved in the study. These potential participants were asked to provide their contact information for the data collection on the day at their convenience. The sampling process continued until we reached information saturation [28].

### Data collection method and procedures

Both in-depth interviews and focus group discussions were used as data collection methods. This method of triangulation was adopted to reveal various dimensions of the phenomenon of interest as suggested by Adam & Kiger [29]. Both in-depth interviews and focus group discussions were conducted in Kiswahili, the most common language used in Tanzania. All interviews and discussions were digitally audio-recorded. Participants were repeatedly reminded that it was at their discretion to stop the interview and leave whenever they felt they needed to do so. Participants received 10,000 Tanzanian Shillings (Tshs) (approximately 5 USD) as compensation for their time and transport.

#### In-depth interview

In-depth interviews were conducted to obtain data from patients with mental illness and healthcare providers. A semi-structured interview guide (S1 Text) was used to interview participants. The questions in the interview guide were developed by the authors through a literature review and modified to fine-tune them during the interview process [30]. The interview guide consisted of questions about the patient and healthcare providers. Each question for patients and healthcare providers was followed up by several probing questions. All Interviews were conducted in rooms located within the hospital premises except the one for the district mental health coordinator which was conducted in the office. The rooms had enough space for free expression and a good sitting arrangement with adequate lighting to easily see non-verbal cues. Moreover, the room was located far away from the patients’ lounges which ensured minimum noise level. Both authors interviewed the participants at different times. Seven patients with mental illness and six healthcare providers were interviewed after attaining information saturation [28]. The interviews lasted between 45 and 60 minutes.

#### Focus group

Focus group discussions were conducted to collect data from caregivers. This method was used to explore common participants’ experiences and beliefs in caring for patients with mental illness. Using group processes, it was possible to stimulate responses and gain insights through participants exchanging views, questioning, and challenging each other [31, 32] on caring activities in the home environment. We adopted a similar interview guide (S2 Text) with questions constructed by the same process as used in in-depth interviews. The guide included questions exploring how caregivers supported patients at home. All focus group discussions were conducted at the hospital premises. The first author moderated the interviews and the second author controlled the external environment and took field notes. Two focus group discussions were conducted each with 8 homogeneous participants. The average duration for focus group discussions was 70 minutes.

### Data analysis

Data analysis began immediately after the first interview to identify emerging patterns and modify interview guidelines for subsequent data collection [33]. However, thorough data analysis was carried out after the completion of data collection. Before commencing analysis, all audio-recorded interviews were reviewed and transcribed verbatim [34]. A person with qualitative research experience helped to transcribe the data. The first author cross-checked the transcripts with the original audio data to ensure correct and accurate information in both versions was provided. A few corrections were made such as typographic errors and some omission of words.

**Fig 1:**
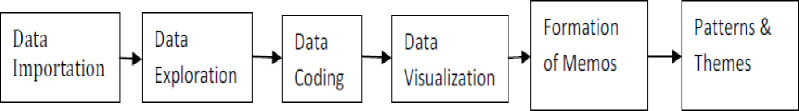
Data analysis leading to theme formation.

Data analysis was performed using the thematic analysis method as described by Braun and Clarke [35]. Interview transcripts and observation notes were systematically used to search for meaning and increase the understanding of the challenges in the context of caring for a patient with mental illness at home. Initially, both authors read the transcripts several times to gain an overall impression of the content and familiarize themselves with the data. Then, parts of transcripts were identified as meaning units and were condensed to form codes. Based on their similarities and differences, the extracted codes were then grouped into categories reflecting the obvious meaning of a text and similar categories were organized into themes reflecting the underlying meaning of a text. This was followed by reviewing and refining potential themes to ensure they were derived from codes. The formed themes were then defined and named, and lastly the process of producing the report was carried out. Analysis was achieved with the use of NVivo 10 software which facilitated organizing data and coding the texts [36]. The coded text was filtered and placed in similar contents that formed a family tree. The identified content of the text was entered into memos which were eventually manually organized into patterns and themes (Fig 1). The unit of analysis was the whole textual data obtained from the in-depth interviews and focus group discussions. Throughout the analysis, the audio data in their entirety served as a point of reference when a deeper understanding of the meaning units, codes, categories, and themes, was required. All steps in the analysis were subjected to discussion with the authors and certain modifications were made following mutual agreement.

### Ethics approval

The ethical approval for this study was obtained from the Directorate of Research and Publication of Muhimbili University of Health and Allied Sciences, with Ref. No. 2015-04-08/AEC/Vol.IX/80. Permission to conduct the study was granted by the Regional Administrative Officer and lower authorities in the district including District Medical Officer. Participants were informed that their involvement in the study would potentially provide the opportunity to gain information that would be used in improving mental health services. Their role in the study as participants was clearly stated and their expectations were clarified. Researchers were flexible to accommodate participants’ needs and variations in availability or attendance. Participants were identified by numbers and not by their names. Written informed consent was sought from each participant before the interview sessions.

## Results

### Participants’ characteristics

Participants’ characteristics are summarized in Table 1. A total of 29 participants participated in the study including 7 patients with severe mental illness, 16 caregivers, and 6 healthcare providers (including 1 District Mental Health Coordinator (DMHC)). The patients interviewed in this study were mainly diagnosed with schizophrenia and bipolar disorders. Years of experience in providing mental health services among healthcare providers ranged from 3 to 18.

**Table 1:** Participants’ characteristics.

Participants		Total number
Patients		7
Caregivers		
	1. Females	8
	2. Males	8
Healthcare providers		
	1. Nurses	3
	2. Assistant medical officers	2
	3. District mental health coordinators	1
Total		29

### Challenges to the provision of home care and support for individuals with severe mental illness

This study unveiled challenges to the provision of home care and support for individuals with severe mental illness. The challenges were categorized under the following four main themes: (1) Poor understanding of mental illness; (2) Abandonment of patients’ care responsibilities; (3) Dispute over preferred treatment modality; (4) Lack of outreach mental health services.

### Challenges related to poor understanding of mental illness

Participants, especially caregivers who did not know the appropriate care to be provided to their patients, acknowledged that they had a poor understanding of mental illness. Caregivers were concerned about living with patients for a long time without understanding their condition. Nevertheless, there was no opportunity provided at the clinic for receiving education about mental illness, although they wanted to learn about causes, prognosis, and home care for individuals with mental illness as described below:

*“For example*, *until now we have stayed with him (patient) for years but we do not know the problem; even if they say it’s schizophrenia*, *I have not understood it*. *I have never been told by anybody regarding what I should do to help the patient (recover) so that*, *maybe*, *one day he will stop using medication*.… *It’s a problem*, *I desire that one day the doctor will tell us what is wrong with our patient and what we should do”* (female caregiver).

Poor mental health knowledge was especially revealed when the patient and caregivers could not recognize symptoms of mental illness the first time the patient became sick. Therefore, they could not take the right decision to seek treatment as stated by a patient:

*“The first time I got this problem was in 2007*. *I think they (family members) did not understand that the problem I had was a mental illness*, *they thought maybe it was malaria*. *So they used to take me to the hospital to have a malaria check-up instead of going to the hospital to get treatment for mental illness*. *Therefore*, *the condition reached a bad stage and I was struggling here and there until I ran away from home and went to stay in another place*, *and the problem was still there*. *It’s a challenge that I experienced”* (male patient with schizophrenia).

The district mental health coordinators reiterated that poor mental health knowledge was a common problem existing in the community, which also contributed to the infringement of the rights of people with mental illness.

*“When people are told about a mentally ill patient*, *it’s like they see it*, *I don’t know how I should say*, *there is very little understanding*, *it is very little in the community to the extent that the community itself does not consider that a person with mental illness can still be having all the rights*. *Therefore*, *knowledge is extremely very little*, *unlike other diseases whose knowledge is well provided*…*”* (district mental health coordinator).

### Challenges related to abandonment of patients’ care responsibilities

Participants, especially caregivers, raised concerns regarding care responsibilities for individuals with mental disorders in the home environment. They stated that some family members often neglected patients and care responsibilities were left to a few members, which created a stressful situation in the family.

*“… The challenge we experience*, *like me here*, *I have a family of many people but it’s like most of them have neglected him (the patient)*. *It is only the remaining three of us who are experiencing these challenges*, *whatever the case*, *to provide care to our patients*. *Only my two sisters*, *provide care to our patients whatever the case*. *Others have despised him and I took it (the mental illness) just as a disease that anybody could get because he (the patient) never wanted to be like this*. *So we are three (those helping the patient); the rest have their issues”* (male caregiver).

Abandonment of care responsibilities by some family members was fueled by various factors such as believing that one can never have a child with mental illness and attributing mental illness to witchcraft. Some female caregivers were left with care responsibilities alone after their husbands abandoned them for such reasons, as verbalized by one of them:

*“I faced a challenge with my patient who happens to be my firstborn child*. *I am separated from his father; his father has abandoned him saying that my mother has bewitched him (the patient)*. *He (the father) has therefore said he will not help him with treatment and if I cannot do so (seek treatment) I should just leave him roaming in the streets since other crazy people roaming in the streets also have relatives”* (male caregiver).

The district mental health coordinator confirmed that patients were abandoned by some caregivers, attributing this problem to a lack of knowledge regarding mental illness among caregivers:

*“… Most people take these people (with mental illness) to the hospital after they have wandered to see traditional healers*, *and at the end of the day*, *they abandon patients and leave them roaming in the streets*, *it is as if they dump them*. *Therefore*, *sometimes the patient with mental illness is brought to the hospital the first*, *second*, *and the third day they leave him struggling alone and this is because the community does not understand how this problem* is*”*. *The main source of the problem could be the family*, *but they are not aware*, *knowledge is inadequate*. *In simple language*, *knowledge is inadequate and is not there”* (district mental health coordinator).

### Challenges related to dispute over preferred treatment modality

Sometimes family members or caregivers disputed over treatment modality that would suit their patient due to differing treatment preferences and had a hard time reaching a consensus. Some preferred traditional treatment while others preferred formal treatment. This was attributed to the influence of culture and tradition and was reported to delay the initiation of patient treatment and worsening of the patient condition as stated by a caregiver:

*"We are also affected by the culture and traditions of our country or we Tanzanians*. *We have no straightforward decisions on the direction we should take*, *you see*. *When we face problems like this (mental illness) we are divided into two sides and we argue*, *some saying we should practice divination and others saying we should go to the hospital*. *So you argue a lot*, *and it can be between the husband and wife; the two of you may argue in the house*, *and by the time you reach a consensus the illness has escalated”* (male caregiver).

Participants also attributed differences in the choice of treatment modality to inadequate knowledge regarding mental illness among caregivers as stated by the district mental health coordinator:

*“… compared to other diseases whose knowledge is well provided*, *knowledge (regarding mental illness) is too little…*. *that’s why many people bring these (mentally sick) people to the hospital after they have wandered seeking help from traditional healers and at the end of the day they leave patients roaming in the streets”* (district coordinator).

### Challenges related to lack of outreach mental health services

Participants acknowledged the need for outreach mental health services to support home care for individuals with mental illness. It was expected that healthcare providers from Temeke hospital would pay regular follow-up home visits to mentally ill individuals to monitor their progress and provide the necessary support, as verbalized by a patient:

*“Maybe*, *for example*, *if they (healthcare providers) visit us where we stay*, *they may discover a problem which is there*. *When mental health people pay home visits and see where you stay*, *they may discover what is needed*. *They will visit so that you stay well at home*. *For example*, *apart from providing medication to a person*, *there are other needs*. *Someone may not be having food*, *so when these (mental health) section workers visit*, *they may discover how to provide help”* (male patient with schizophrenia).

Caregivers also stated the role of outreach services in patient follow-up and that it was important for coordination between hospital and home care, although they reported that it was not happening.

*“Concerning home (care)*, *I agree with the others that there should be follow-up*. *Let me say that there should be coordination between care received at the hospital and home*. *Not that they just listen to him (a patient) when he comes to the hospital and say how are you doing*, *I don’t know*, *they prescribe him and he leaves*. *I believe that medications are not the only solution*. *… It is true; there is no coordination between hospital services and home care*. *We do what we can to do*, *whether it is nutrition*, *whether it is anything we are supposed to do*, *or whether it is the medication*, *we buy*. *You will try to do everything*, *you will provide counseling*, *but you do not have that expertise*, *you see …”* (female caregiver).

Healthcare providers stated that outreach mental health services were not carried out for a long time as stated by a social worker who also emphasized its importance but was not sure when it would be available.

*“There are no outreach services*. *However*, *those patients with mental illness need to be visited at home*. *In Temeke (district)*, *it has never happened and I do not know whether it will ever happen*, *I do not know*, *maybe after I have retired*. *We are seeking to visit patients*, *some of whom are in difficult situations but there are no outreach services*, *and outreach services in mental health are very important”* (female social worker).

The district mental health coordinator reiterated the lack of outreach services for individuals with mental disorders and mentioned various resource-related reasons and challenges experienced with its implementation including budget and transport issues:

*“We do not yet have strategies (to make a follow-up)*, *it happens at the family level because when they (patients) wander around*, *they are brought to the hospital by their family*. *However*, *we also cannot track them due to lack of transport*, *though we might have the mapping to know where they stay*. *Now how do you follow them; you need transport*. *You fail because there is no transport*, *but they (patients) are many”* (district mental health coordinator).

### Suggestions to improve home care and support

Apart from stating challenges, participants suggested key areas and strategies through which home care and support for individuals with mental illness can be improved. These included establishing a mental health care support call center, setting a special day at the mental health clinic for the provision of health education, providing health education on mental health to decision-makers and people in the community, and establishing rehabilitation centers for patients with mental illness.

Establishing a mental healthcare support call center in the district was suggested as a means which caregivers could use to communicate with mental healthcare providers on various issues such as transport for patients during emergencies as stated by a caregiver:

*“… If there was a section there (at the hospital)*, *we would be allowed to make calls that I have a mentally ill patient*. *Then*, *if there is support*, *a car could be brought with a health expert*, *and working together with community members they can even sedate the patient with medications so that (the patient) arrives here (at the clinic) in a good condition*, *rather than struggling by ourselves*, *we sometimes fail*. *Even at a lower price*, *we can share the cost for bringing the ambulance*, *at least at a small percentage*, *instead of taking a taxi driver and paying for the car windows which are broken (due to aggressive behavior of patients)”* (male caregiver).

The suggestion of setting a special day at the mental health clinic for providing health education was based on the need for caregivers to understand the patient’s condition and receive advice so that appropriate care is provided as stated by a caregiver.

*“*… . *a special day could be set*, *even if it’s after every three months*, *to meet with our nurses and doctors who see our patients like the way we are seated here (in an FGD)*, *you see*. *We exchange views and they advise us on how we should handle our patients so that we do not make mistakes*. *They should also give us advice and tell us these patients*, *you know*, *need great patience since sometimes they become harsh and this …”* (female caregiver).

Participants suggested the need for providing health education to decision-makers and people in the community. This is because decision-makers at the hospital did not provide support to healthcare providers to carry out outreach services, and due to poor mental health knowledge in the community.

*“The first thing is that decision-makers should be given education so that they understand the meaning of mental health*, *because if decision-makers are not educated*. *They will just be surprised when you give them the budget (for mental health services) and they will not understand why these people (healthcare providers) need the budget”* (female nurse and social worker).

*“*… *the first thing to do I think*, *is health education to the community so that they may understand what causes mental disorders*, *especially for those caused by substance use like marijuana*, *so that the community is educated on its adverse effects”* (female nurse).

Lastly, participants suggested establishing rehabilitation centers that could serve patients recovering from chronic mental illness and preparing them to be self-reliant as stated by a healthcare provider:

*“There should also be rehabilitation centers*, *if a patient had a mental illness for a long time and is not involved in any self-reliant activity even after recovering*, *there should be at least a place to prepare him to become self-reliant after recovery*.*”* (female nurse).

## Discussion

This study aimed to explore challenges to home care and support for people with mental illness. Findings revealed five main themes, four highlighting challenges in the provision of home care and support for individuals with mental illness, and one suggesting an improvement to home care and support for people with mental illness. The four themes highlighting challenges in the provision of home care and support were poor understanding of mental illness, abandonment of patients’ care responsibilities, the dispute over preferred treatment modality, and lack of outreach mental health services.

Poor understanding of mental illness among caregivers was identified as one of the setbacks to the provision of home care and support for individuals with mental illness. This highlights not only low mental health awareness in the community but also the lack of opportunities for educational support among caregivers during clinic visits. Poor knowledge regarding mental health is a common phenomenon in low and middle-income countries [37–39]. This underscores the need for community mental health awareness programs to improve mental health literacy [40]. To achieve this, various strategies may be employed such as whole-of-community campaigns and participation in family-led education interventions, which may provide families with the information they need to better cope with their relative’s mental illness [41, 42].

Abandonment of patients’ care responsibilities was another key finding that affected the provision of care and support to individuals with mental illness, sometimes leading to a marriage breakup. This was fueled by cultural practices such as beliefs in witchcraft and a misconception that one’s family can never develop mental illness. This again underscores the lack of mental health knowledge in the community as previously discussed [37–39]. Rejection and abandonment of patients’ care responsibilities in families have been previously reported and are attributed to multiple reasons. These include helpless abandonment (inability to meet patient’s needs due to poverty), careless abandonment (not being ready to shoulder the caregiving responsibility of the person with mental illness), and willful abandonment (intentional dumping of the mentally ill family member by other significant relatives due to vested interests such and getting divorce and property) which may all lead to homelessness [43]. Marriage breakups or divorce because of the stigma associated with mental illness has also been previously reported [44]. Therefore, mental health education and stigma reduction programs in the community may be useful in addressing the abandonment of patients with mental illness [45].

Some caregivers in this study experienced disputes over the preferred treatment modality for their patients, which delayed the initiation of proper treatment. This shows different health beliefs and views individuals may hold about mental illness, not only in the community but also at the family level. A previous study in Tanzania reported a lack of harmony among caregivers caused by disagreement on the right treatment for the patient, as one party wanted to see a traditional healer while the other wanted to seek spiritual help [46]. Culture and context are said to have a profound effect on the entire help-seeking pathway for mental illness, from problem identification to the choice of treatment providers [47]. Beliefs about mental illness and various magico-religious attributions are one of the reasons that delay patients’ treatment [48]. For instance, caregivers may spend a lot of time seeing a traditional healer with limited knowledge about mental illness leading to delays in treatment for their patient. That’s why there is a need, not only to educate caregivers about mental illness but also for traditional healers to recognize different types of mental disorders and make referrals when patients are not responding to their mode of treatment [49]. This implies that mental healthcare providers need to work in collaboration with other service providers in the community such as traditional healers and religious leaders because whether we like it or not, patients and families will always seek care from them [50–52]. Literature suggests that alternative practitioners, including traditional healers, appear to play an important role in the delivery of mental health services [53]. The collaboration between mental health professionals and alternative providers might help in minimizing conflicts in families that emanate from treatment preferences. There is also a need to provide social support to caregivers and promote resilience factors to cope with the situation of living with a mentally ill person [54, 55].

Home visits for people with mental illness play an important role in monitoring their progress and providing the necessary support and are associated with improved or maintained psychiatric status [10, 56]. However, participants in the current study verbalized a lack of outreach mental health services by healthcare providers to support home care due to a lack of resources (budget and transport). A similar situation has been reported in Kenya where the budget for mental health supervisory activities was not included in the overall health supervision budget, highlighting priority challenges in implementing community mental health services in low-income settings [57, 58]. The right approach to ensure the availability of funds for mental health as stipulated by WHO is to include the budget within general health financing [40]. This stresses the need for prioritization of mental health services which can be achieved only through political will and strengthened legislation, improved resource allocation and strategic organization, integrated packages of care, and involvement of patients, informal healthcare providers, and the wider community in a therapeutic capacity [17].

Participants in the current study suggested establishing a mental health care support call center, setting a special day at the mental health clinic for the provision of health education, providing health education on mental health to decision-makers and people in the community, and establishing rehabilitation centers for patients with mental illness as the support needed for people with mental illness. Most of these suggestions corroborate with evidence-based service needs for people with mental illness [9, 59, 60]. Moreover, the suggestions highlight challenges regarding the availability and accessibility of such support services. For instance, studies show that evidence-based rehabilitative interventions for people with severe mental illness are not widely available in real-world practice [61], stressing the existing treatment gap, especially in low-income settings where there are limited material and human resources [62].

As a qualitative study using purposive sampling, our findings may not apply across the study populations in Tanzania; our intention was to understand in-depth rather than to generalize. In particular, the study samples may be more representative of challenges to home care and support for people with mental illness in the study settings. Also, sensitive and personal information could not be shared in the focus group discussions, which might have affected the results. Finally, this study involved patients, caregivers, and healthcare providers. Other important stakeholders such as community members and the hospital management team were not included. Their involvement would provide more insight into the challenges experienced in providing home care and support for people with mental illness. However, the information provided by the participants is useful as we wanted to explore the shared challenges to provision of home care and support for people with severe mental illness in the local context.

## Conclusion

This study reveals a poor understanding of mental illness, abandonment of patients’ care responsibilities, disputes over preferred treatment modality, and lack of outreach to mental health services as challenges experienced in the provision of home care and support for people with mental illness. Caregivers’ mental illness education, stigma reduction programs, funding and prioritization of outreach home services, and improving communication between healthcare providers and families with patients with mental illness are important in improving home care and support for people with mental illness. Our study suggests higher authorities such as national, regional, and district mental health coordinators to collaboratively work with the healthcare providers to improve the provision of services in the home environment for the wellbeing of patients with severe mental illness. Further research is needed to determine the magnitude of these challenges and factors that can facilitate the provision of support in similar settings.

## Supporting information

S1 Text A guide for in-depth interview. (PDF)

S2 Text A guide for focus group discussion. (PDF)

S3 Text Excerpts of transcripts. (PDF)
