# Supplementary material for: Challenges to the provision of home care and support for people with severe mental illness: Experiences and perspectives of patients, caregivers, and healthcare providers in Dar es Salaam, Tanzania
Source: PLOS Glob Public Health. 2023 Jan 24;3(1):e0001518. doi: 10.1371/journal.pgph.0001518 (PMC10021743; doi:10.1371/journal.pgph.0001518)
Supplement: S1 Text — (PDF) [file pgph.0001518.s001.pdf]

The guide for in-depth interview (IDI)

For patients with mental illness;

1. what support do you receive from family members as far as your mental illness is concerned?
2. What challenges do you face in getting this support?
3. What kind of support do you think is important for you to receive from people close to you at home about your mental illness?
4. What kind of support do you receive from people in the community?
5. What challenges do you face with people in the community?

For healthcare providers;

1. What is your experience in providing mental health services to patients and the community? (Probe on type, quality, and accessibility of services)
2. What training did you receive to provide mental health care to patients? (Probe level of education, specialization, short course or on-the-job training)
3. What do you think should be done to improve services for patients with mental illness?
