# Supplementary material for: Challenges to the provision of home care and support for people with severe mental illness: Experiences and perspectives of patients, caregivers, and healthcare providers in Dar es Salaam, Tanzania
Source: PLOS Glob Public Health. 2023 Jan 24;3(1):e0001518. doi: 10.1371/journal.pgph.0001518 (PMC10021743; doi:10.1371/journal.pgph.0001518)
Supplement: S2 Text — (PDF) [file pgph.0001518.s002.pdf]

The guide for focus group discussion (FGD)

1. What do you think are the needs of a mentally ill patient at home? (Probe needs during crisis, financial support, psychosocial support etc)
2. What are your views about how these needs are met?
3. What support have you been providing to the patients with mental illness in your homes and what are the challenges faced?
4. What would be the best way to provide support to the mentally ill individual at home?
