# Supplementary material for: Challenges to the provision of home care and support for people with severe mental illness: Experiences and perspectives of patients, caregivers, and healthcare providers in Dar es Salaam, Tanzania
Source: PLOS Glob Public Health. 2023 Jan 24;3(1):e0001518. doi: 10.1371/journal.pgph.0001518 (PMC10021743; doi:10.1371/journal.pgph.0001518)
Supplement: S3 Text — (PDF) [file pgph.0001518.s003.pdf]

Challenges to home care and support for people with mental illness: Experiences and perspectives of patients, caregivers, and healthcare providers in Dar es Salaam, Tanzania
Sociodemographic characteristics data

Demographic data

Participants	Total number
Patients	7
Caregivers	
1. Females	8
2. Males	8
Healthcare providers	
1. Nurses	3
2. Assistant medical officers	2
3. District mental health coordinators	1
Total	29

Excerpts of the transcripts

Poor understanding of mental illness

"For example, until now we have stayed with him (patient) for years but we do not know the problem, even if they say it's a disorder failure, I have not understood. I have never been told by anybody that you should do one two three so that maybe one day he will stop using medication or so because I see that medications help him. ... It's a problem, I desire that one day the doctor will tell us what is wrong with our patient and what we should do." (FGD 1, CG No. 4)

"The first time I got this problem was in 2007. I think they (family members) did not understand that the problem I had was a mental illness, they thought maybe it was malaria. So they used to take me to the hospital to have a malaria check-up instead of going to the hospital to get treatment for mental illness. Therefore, the condition reached a bad stage and I was struggling here and there until I ran away from home and went to stay in another place, and the problem was still there. It's a challenge that I experienced." (Pt with schizophrenia)

"When people are told about a mentally ill patient, it's like they see it, I don't know how I should say, there is very little understanding, it is very little in the community to the extent that the community itself does not consider that a person with mental illness can still be having all the rights. Therefore, knowledge is extremely very little, unlike other diseases whose knowledge is well provided ..." (District Coordinator)

Abandonment of patients' care responsibilities

"... The challenge we experience, like me here, I have a family of many people but it's like most of them have neglected him (the patient). It is only the remaining three of us who are experiencing these challenges, whatever the case, to provide care to our patients. Only my two sisters, provide care to our patients whatever the case. Others have despised him and I took it (the mental illness) just as a disease that anybody could get because he (the patient) never wanted to be like this. So we are three (those helping the patient); the rest have their issues." (FGD 2, CG No. 8)

"I faced a challenge with my patient who happens to be my firstborn child. I am separated from his father; his father has abandoned him saying that my mother has bewitched him (the patient). He (the father) has therefore said he will not help him with treatment and if I cannot do so (seek treatment) I should just leave him roaming in the streets since other crazy people roaming in the streets also have relatives." (FGD 2, CG No. 1).

"... Most people take these people (with mental illness) to the hospital after they have wandered to see traditional healers, and at the end of the day, they abandon patients and leave them roaming in the streets, it is as if they dump them. Therefore, sometimes the patient with mental illness is brought to the hospital the first, second, and the third day they leave him struggling alone and this is because the community does not understand how this problem is*". The main source of the problem could be the family, but they are not aware, knowledge is inadequate. In simple language, knowledge is inadequate and is not there."* (DMHC)

Dispute over preferred treatment modality

"We are also affected by the culture and traditions of our country or we Tanzanians. We have no straightforward decisions on the direction we should take, you see. When we face problems like this (mental illness) we are divided into two sides and we argue, some saying we should practice divination and others saying we should go to the hospital. So you argue a lot, and it can be between the husband and wife; the two of you may argue in the house, and by the time you reach a consensus the illness has escalated." (FGD 2 CG No. 10).

"... compared to other diseases whose knowledge is well provided, knowledge (regarding mental illness) is too little.... that's why many people bring these (mentally sick) people to the hospital after they have wandered seeking help from traditional healers and at the end of the day they leave patients roaming in the streets." (DMHC)

Lack of outreach mental health services

"Maybe, for example, if they (healthcare providers) visit us where we stay, they may discover a problem which is there. When mental health people pay home visits and see where you stay, they may discover what is needed. They will visit so that you stay well at home. For example, apart from providing medication to a person, there are other needs. Someone may not be having food, so when these (mental health) section workers visit, they may discover how to provide help." (Pt with schizophrenia)

"Concerning home (care), I agree with the others that there should be follow-up. Let me say that there should be coordination between care received at the hospital and home. Not that they just listen to him (a patient) when he comes to the hospital and say how are you doing, I don't know, they prescribe him and he leaves. I believe that medications are not the only solution. ... It is true; there is no coordination between hospital services and home care. We do what we can to do, whether it is nutrition, whether it is anything we are supposed to do, or whether it is the medication, we buy. You will try to do everything, you will provide counseling, but you do not have that expertise, you see ..." (FGD 1, CG No. 4)

"There are no outreach services. However, those patients with mental illness need to be visited at home. In Temeke (district), it has never happened and I do not know whether it will ever happen, I do not know, maybe after I have retired. We are seeking to visit patients, some of whom are in difficult situations but there are no outreach services, and outreach services in mental health are very important." (Social Worker)

"We do not yet have strategies (to make a follow-up), it happens at the family level because when they (patients) wander around, they are brought to the hospital by their family. However, we also cannot track them due to lack of transport, though we might have the mapping to know where they stay. Now how do you follow them; you need transport. You fail because there is no transport, but they (patients) are many." (DMHC)

Suggestions to improve home care and support

"… If there was a section there (at the hospital), we would be allowed to make calls that I have a mentally ill patient. Then, if there is support, a car could be brought with a health expert, and working together with community members they can even sedate the patient with medications so that (the patient) arrives here (at the clinic) in a good condition, rather than struggling by ourselves, we sometimes fail. Even at a lower price, we can share the cost for bringing the ambulance, at least at a small percentage, instead of taking a taxi driver and paying for the car windows which are broken (due to aggressive behavior of patients)." (FGD 2 CG No 10)

".... a special day could be set, even if it's after every three months, to meet with our nurses and doctors who see our patients like the way we are seated here (in an FGD), you see. We exchange views and they advise us on how we should handle our patients so that we do not make mistakes. They should also give us advice and tell us these patients, you know, need great patience since sometimes they become harsh and this …" (FGD 1 CG NO. 3)

"The first thing is that decision-makers should be given education so that they understand the meaning of mental health, because if decision-makers are not educated. They will just be surprised when you give them the budget (for mental health services) and they will not understand why these people (healthcare providers) need the budget" (HC SW)

"… the first thing to do I think, is health education to the community so that they may understand what causes mental disorders, especially for those caused by substance use like marijuana, so that the community is educated on its adverse effects." (HC Nurse)

"There should also be rehabilitation centers, if a patient had a mental illness for a long time and is not involved in any self-reliant activity even after recovering, there should be at least a place to prepare him to become self-reliant after recovery." (HC Nurse)
